# Aqua­bis­(4-chloro-2-hy­droxy­benzoato-κ*O*)(1,10-phenanthroline-κ^2^ *N*,*N*′)zinc(II)

**DOI:** 10.1107/S1600536811020435

**Published:** 2011-06-04

**Authors:** Jing-Jing Nie, Xun Xu, Duan-Jun Xu

**Affiliations:** a Department of Chemistry, Zhejiang University, People’s Republic of China; b Department of Electrical Engineering and Information Technology, Faculty of Engineering, Kyushu Sangyo University, Japan

## Abstract

In the title compound, [Zn(C_7_H_4_ClO_3_)_2_(C_12_H_8_N_2_)(H_2_O)], the Zn^II^ cation is coordinated by two 4-chloro-2-salicylate anions, one 1,10-phenanthroline ligand and one water mol­ecule in a square-pyramidal coordination geometry; the Zn cation lies 0.4591 (11) Å from the basal plane. The benzene rings of the anions are involved in π–π stacking. The centroid–centroid distance between parallel benzene rings of adjacent mol­ecules is 3.9017 (17) Å, and the centroid–centroid distance between benzene and pyridine rings of adjacent mol­ecules is 3.584 (2) Å. Intra­molecular O—H⋯O hydrogen bonding is present.

## Related literature

For general background on π–π stacking, see: Deisenhofer & Michel (1989 ▶). For π–π stacking in dihy­droxy­benzoate complexes, see: Yang *et al.* (2006 ▶); Zhang *et al.* (2008 ▶). For π–π stacking found in chloro­benzoate complexes, see: Maroszová *et al.* (2006 ▶); Malamatari *et al.* (1995 ▶); Wen & Ying (2007 ▶); Wen *et al.* (2007 ▶). For centroid-to-centroid distances between benzene rings in salicylate complexes, see: Allen (2002 ▶).
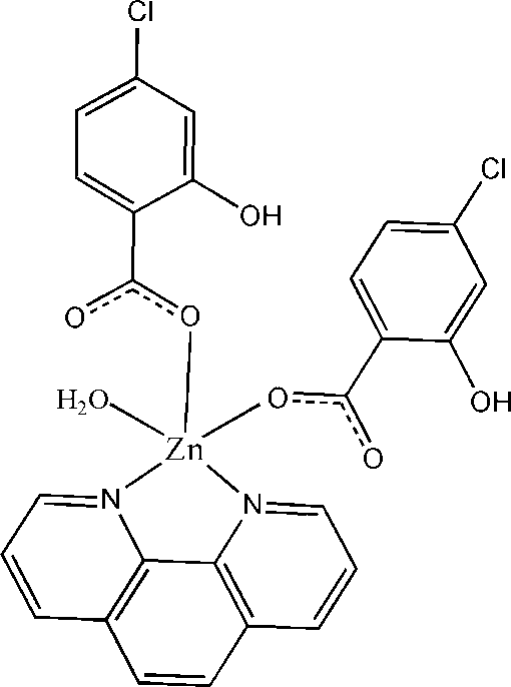


## Experimental

### 

#### Crystal data

[Zn(C_7_H_4_ClO_3_)_2_(C_12_H_8_N_2_)(H_2_O)]
*M* *_r_* = 606.69
Triclinic, 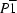

*a* = 8.2611 (12) Å
*b* = 11.0124 (16) Å
*c* = 14.654 (2) Å
α = 100.534 (7)°
β = 94.360 (8)°
γ = 111.315 (5)°
*V* = 1206.1 (3) Å^3^
*Z* = 2
Mo *K*α radiation
μ = 1.29 mm^−1^
*T* = 294 K
0.28 × 0.20 × 0.12 mm

#### Data collection

Rigaku R-AXIS RAPID IP diffractometer
Absorption correction: multi-scan (*ABSCOR*; Higashi, 1995 ▶) *T*_min_ = 0.86, *T*_max_ = 0.92
13131 measured reflections
4275 independent reflections
3695 reflections with *I* > 2σ(*I*)
*R*_int_ = 0.027

#### Refinement

*R*[*F* ^2^ > 2σ(*F* ^2^)] = 0.034
*wR*(*F* ^2^) = 0.101
*S* = 1.05
4274 reflections
343 parameters
H-atom parameters constrained
Δρ_max_ = 0.64 e Å^−3^
Δρ_min_ = −0.29 e Å^−3^

### 

Data collection: *PROCESS-AUTO* (Rigaku, 1998 ▶); cell refinement: *PROCESS-AUTO*; data reduction: *CrystalStructure* (Rigaku/MSC, 2002 ▶); program(s) used to solve structure: *SIR92* (Altomare *et al.*, 1993 ▶); program(s) used to refine structure: *SHELXL97* (Sheldrick, 2008 ▶); molecular graphics: *ORTEP-3 for Windows* (Farrugia, 1997 ▶); software used to prepare material for publication: *WinGX* (Farrugia, 1999 ▶).

## Supplementary Material

Crystal structure: contains datablock(s) I, global. DOI: 10.1107/S1600536811020435/ng5174sup1.cif

Structure factors: contains datablock(s) I. DOI: 10.1107/S1600536811020435/ng5174Isup2.hkl

Additional supplementary materials:  crystallographic information; 3D view; checkCIF report

## Figures and Tables

**Table 1:** Selected bond lengths (Å)

Zn—O1	2.0155 (18)
Zn—O4	2.0325 (19)
Zn—O7	2.109 (2)
Zn—N1	2.130 (2)
Zn—N2	2.126 (2)

**Table 2:** Hydrogen-bond geometry (Å, °)

*D*—H⋯*A*	*D*—H	H⋯*A*	*D*⋯*A*	*D*—H⋯*A*
O3—H3*A*⋯O2	0.95	1.66	2.559 (3)	155
O6—H6*A*⋯O5	0.95	1.72	2.595 (3)	151
O7—H7*A*⋯O2	0.86	1.93	2.707 (3)	150
O7—H7*B*⋯O5	0.96	1.75	2.674 (3)	163

**Fig. 1.**
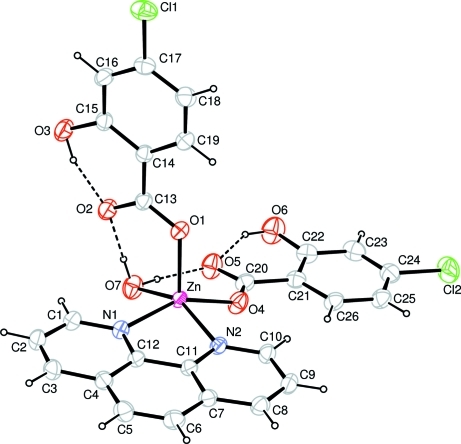
The molecular structure of the title compound with 30% probability displacement ellipsoids. Dashed lines indicate O—H···O hydrogen bonds.

**Fig. 2.**
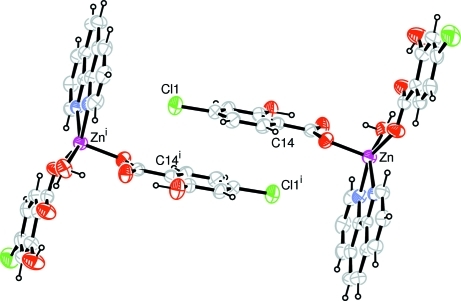
A diagram showing π-π stacking between parallel chls ligands, [symmetry code: (i) 1 - x, 1 - y, 2 - z].

**Fig. 3.**
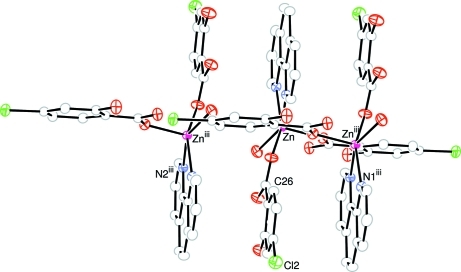
A diagram showing π-π stacking between chls and phen ligands [symmetry codes: (ii) 2 - x, 1 - y, 1 - z; (iii) 1 - x, 1 - y, 1 - z].

## supplementary crystallographic information

### Comment

As π-π stacking between aromatic rings plays an important role in the electron transfer process in some biological system (Deisenhofer & Michel, 1989), the π-π stacking has attracted our much attention in past years. In order to understand the nature of π-π stacking between aromatic rings, we have determined crystal structures of metal complexes with aromatic ligands to investigate the factors controlling aromatic stacking.

Our previous studies on dihydroxybenzoate complexes has revealed that hydroxy-substitution of the aromatic ring may be an effective factor for π-π stacking (Yang *et al.*, 2006; Zhang *et al.*, 2008). As a continued investigation, the title chlorine-substituted salicylate complex has been prepared in the laboratory and its crystal structure is presented here to show the effect of chlorine-substitution on π-π stacking between benzene rings of chlorine-substituted salicylates.

The molecular structure of the title compound is shown in Fig. 1. The Zn(II) cation is coordinated by one phenanthroline (phen) ligand, two chloro-salicylate (chls) anions and one water molecule in a distorted square-pyramidal coordination geometry (Table 1). The Zn atom is 0.4591 (12) Å deviated from the basal plane towards the apical O1 atom. Uncoordinated carboxyl oxygen atoms, O2 and O5, are simultaneously hydrogen bonded to the coordinated water molecule and hydroxyl group of the same chls anion (Table 2).

It is notable π-π stacking between benzene rings of chls anions in the crystal structure. A partially overlapped arrangement is observed between parallel chls anions of neighboring complexes (Fig. 2). The face-to-face separation between C14-benzene ring C14^i^-benzene ring is 3.449 (3) Å, and the centroid-to-centroid distance is 3.9003 (17) Å [symmetry code: (i) 1 - *x*, 1 - *y*, 2 - *z*]. These facts clearly indicate the existence of aromatic stacking between benzene rings of chls anions.

A partially overlapped arrangement is also observed between nearly parallel chls anion and phen ligands of neighboring complexes (Fig. 3). The centroid-to-centroid separation between C26-benzene and N1^ii^-phen is 3.5841 (18) Å [symmetry code: (ii) 2 - *x*, 1 - *y*, 1 - *z*], and that between C26-benznen and N2^iii^-phen is 3.584 (2) Å [symmetry code: (iii) 1 - *x*, 1 - *y*, 1 - *z*]. These findings also suggest that the chls is involved in π-π stacking in the crystal structure; similar to that found in reported metal complexes with chls ligands (Maroszová *et al.*, 2006; Malamatari *et al.*, 1995; Wen & Ying, 2007; Wen *et al.*, 2007).

As π-π stacking interaction does not occur between benzene ring in salicylate complexes (Allen, 2002), but occurs in the chloro-salicylate complex. This reveals the effect of chloro-substitution on aromatic π-π stacking.

### Experimental

An ethanol solution (10 ml) of 1,10-phenanthroline (0.200 g, 1 mmol) was slowly added to an aqueous solution (5 ml) containing Zn(NO_3_)_2_.6H_2_O (0.300 g, 1 mmol), 4-chloro-salicylic acid (0.170 g, 1 mmol) and Na_2_CO_3_ (0.053 g, 0.5 mmol) with continuous stirring. The above reaction mixture was refluxed for 4 h. After cooling to room temperature the solution was filtered. Single crystals were obtained from the filtrate by slow vaporization of solvent after 3 d.

### Refinement

In the final cycles of refinement, a reflection (001) was omitted. Water and hydroxyl H atoms were located in a difference Fourier map and refined as riding in their as-found relative positions with *U*_iso_(H) = 1.5*U*_eq_(O). Aromatic H atoms were placed in calculated positions with C—H = 0.93 Å and refined in riding mode with *U*_iso_(H) = 1.2*U*_eq_(C).

### Crystal data

**Table:**

[Zn(C_7_H_4_ClO_3_)_2_(C_12_H_8_N_2_)(H_2_O)]	*Z* = 2
*M**_r_* = 606.69	*F*(000) = 616
Triclinic, *P*1	*D*_x_ = 1.671 Mg m^−^^3^
Hall symbol: -P 1	Mo *K*α radiation, λ = 0.71073 Å
*a* = 8.2611 (12) Å	Cell parameters from 4275 reflections
*b* = 11.0124 (16) Å	θ = 1.4–25.2°
*c* = 14.654 (2) Å	µ = 1.29 mm^−^^1^
α = 100.534 (7)°	*T* = 294 K
β = 94.360 (8)°	Prism, yellow
γ = 111.315 (5)°	0.28 × 0.20 × 0.12 mm
*V* = 1206.1 (3) Å^3^	

### Data collection

**Table:**

Rigaku R-AXIS RAPID IP diffractometer	4275 independent reflections
Radiation source: fine-focus sealed tube	3695 reflections with *I* > 2σ(*I*)
graphite	*R*_int_ = 0.027
Detector resolution: 10.00 pixels mm^-1^	θ_max_ = 25.2°, θ_min_ = 1.4°
ω scans	*h* = −9→9
Absorption correction: multi-scan (*ABSCOR*; Higashi, 1995)	*k* = −13→13
*T*_min_ = 0.86, *T*_max_ = 0.92	*l* = −17→17
13131 measured reflections	

### Refinement

**Table:**

Refinement on *F*^2^	Primary atom site location: structure-invariant direct methods
Least-squares matrix: full	Secondary atom site location: difference Fourier map
*R*[*F*^2^ > 2σ(*F*^2^)] = 0.034	Hydrogen site location: inferred from neighbouring sites
*wR*(*F*^2^) = 0.101	H-atom parameters constrained
*S* = 1.05	*w* = 1/[σ^2^(*F*_o_^2^) + (0.0614*P*)^2^ + 0.2083*P*] where *P* = (*F*_o_^2^ + 2*F*_c_^2^)/3
4274 reflections	(Δ/σ)_max_ = 0.001
343 parameters	Δρ_max_ = 0.64 e Å^−^^3^
0 restraints	Δρ_min_ = −0.29 e Å^−^^3^

### Special details

**Table:**

Geometry. All e.s.d.'s (except the e.s.d. in the dihedral angle between two l.s. planes) are estimated using the full covariance matrix. The cell e.s.d.'s are taken into account individually in the estimation of e.s.d.'s in distances, angles and torsion angles; correlations between e.s.d.'s in cell parameters are only used when they are defined by crystal symmetry. An approximate (isotropic) treatment of cell e.s.d.'s is used for estimating e.s.d.'s involving l.s. planes.
Refinement. Refinement of *F*^2^ against ALL reflections. The weighted *R*-factor *wR* and goodness of fit *S* are based on *F*^2^, conventional *R*-factors *R* are based on *F*, with *F* set to zero for negative *F*^2^. The threshold expression of *F*^2^ > σ(*F*^2^) is used only for calculating *R*-factors(gt) *etc*. and is not relevant to the choice of reflections for refinement. *R*-factors based on *F*^2^ are statistically about twice as large as those based on *F*, and *R*- factors based on ALL data will be even larger.

### Fractional atomic coordinates and isotropic or equivalent isotropic displacement parameters (Å^2^)

**Table:**

	*x*	*y*	*z*	*U*_iso_*/*U*_eq_	
Zn	0.82980 (4)	0.50277 (3)	0.61362 (2)	0.03699 (12)	
Cl1	0.11084 (11)	0.33056 (8)	1.01904 (6)	0.0632 (2)	
Cl2	0.18339 (13)	0.01494 (10)	0.08427 (6)	0.0760 (3)	
N1	1.0170 (3)	0.6673 (2)	0.71593 (15)	0.0386 (5)	
N2	0.8102 (3)	0.6686 (2)	0.56568 (14)	0.0345 (5)	
O1	0.6382 (2)	0.44332 (18)	0.69227 (12)	0.0418 (4)	
O2	0.7884 (3)	0.3686 (2)	0.78612 (13)	0.0474 (5)	
O3	0.6810 (3)	0.3089 (2)	0.93748 (14)	0.0553 (5)	
H3A	0.7437	0.3201	0.8855	0.083*	
O4	0.7237 (3)	0.39070 (18)	0.48131 (13)	0.0505 (5)	
O5	0.7478 (3)	0.1972 (2)	0.48595 (15)	0.0586 (6)	
O6	0.5411 (4)	−0.0177 (2)	0.36472 (17)	0.0726 (7)	
H6A	0.6098	0.0407	0.4219	0.109*	
O7	0.9615 (3)	0.3763 (2)	0.63633 (14)	0.0550 (5)	
H7A	0.9124	0.3467	0.6815	0.082*	
H7B	0.8986	0.3023	0.5846	0.082*	
C1	1.1169 (4)	0.6657 (3)	0.7912 (2)	0.0468 (7)	
H1	1.1155	0.5836	0.7996	0.056*	
C2	1.2232 (4)	0.7806 (3)	0.8579 (2)	0.0513 (8)	
H2	1.2903	0.7748	0.9096	0.062*	
C3	1.2277 (4)	0.9020 (3)	0.8463 (2)	0.0484 (7)	
H3	1.2990	0.9797	0.8901	0.058*	
C4	1.1245 (3)	0.9098 (3)	0.76815 (18)	0.0398 (6)	
C5	1.1198 (4)	1.0317 (3)	0.7507 (2)	0.0472 (7)	
H5	1.1891	1.1122	0.7923	0.057*	
C6	1.0167 (4)	1.0327 (3)	0.6750 (2)	0.0469 (7)	
H6	1.0161	1.1137	0.6653	0.056*	
C7	0.9084 (3)	0.9110 (3)	0.60930 (18)	0.0376 (6)	
C8	0.7985 (4)	0.9050 (3)	0.5286 (2)	0.0440 (7)	
H8	0.7930	0.9831	0.5157	0.053*	
C9	0.7007 (4)	0.7849 (3)	0.46974 (19)	0.0438 (7)	
H9	0.6292	0.7805	0.4160	0.053*	
C10	0.7084 (4)	0.6680 (3)	0.49066 (18)	0.0398 (6)	
H10	0.6394	0.5863	0.4503	0.048*	
C11	0.9101 (3)	0.7889 (2)	0.62411 (17)	0.0321 (5)	
C12	1.0201 (3)	0.7880 (2)	0.70504 (17)	0.0335 (6)	
C13	0.6586 (4)	0.3973 (2)	0.76426 (18)	0.0369 (6)	
C14	0.5204 (3)	0.3775 (2)	0.82579 (17)	0.0339 (6)	
C15	0.5405 (4)	0.3348 (3)	0.90948 (18)	0.0380 (6)	
C16	0.4128 (4)	0.3204 (3)	0.96834 (19)	0.0435 (7)	
H16	0.4265	0.2934	1.0240	0.052*	
C17	0.2668 (4)	0.3462 (3)	0.9437 (2)	0.0434 (7)	
C18	0.2414 (4)	0.3856 (3)	0.8610 (2)	0.0471 (7)	
H18	0.1410	0.4014	0.8448	0.056*	
C19	0.3681 (4)	0.4009 (3)	0.8035 (2)	0.0424 (6)	
H19	0.3521	0.4276	0.7480	0.051*	
C20	0.6860 (4)	0.2680 (3)	0.44804 (19)	0.0404 (6)	
C21	0.5611 (4)	0.2044 (3)	0.35760 (18)	0.0398 (6)	
C22	0.4962 (4)	0.0653 (3)	0.3208 (2)	0.0469 (7)	
C23	0.3784 (4)	0.0078 (3)	0.2366 (2)	0.0529 (8)	
H23	0.3348	−0.0841	0.2125	0.063*	
C24	0.3277 (4)	0.0890 (3)	0.1900 (2)	0.0494 (7)	
C25	0.3862 (4)	0.2253 (3)	0.2244 (2)	0.0488 (7)	
H25	0.3482	0.2781	0.1925	0.059*	
C26	0.5025 (4)	0.2811 (3)	0.30724 (18)	0.0435 (7)	
H26	0.5438	0.3732	0.3306	0.052*	

### Atomic displacement parameters (Å^2^)

**Table:**

	*U*^11^	*U*^22^	*U*^33^	*U*^12^	*U*^13^	*U*^23^
Zn	0.0433 (2)	0.03601 (18)	0.03246 (19)	0.01538 (14)	0.00390 (13)	0.01019 (13)
Cl1	0.0549 (5)	0.0669 (5)	0.0653 (5)	0.0179 (4)	0.0281 (4)	0.0126 (4)
Cl2	0.0702 (6)	0.0862 (6)	0.0459 (5)	0.0146 (5)	−0.0049 (4)	−0.0093 (4)
N1	0.0374 (12)	0.0438 (12)	0.0362 (12)	0.0151 (10)	0.0039 (9)	0.0149 (10)
N2	0.0395 (12)	0.0365 (11)	0.0283 (11)	0.0144 (9)	0.0053 (9)	0.0100 (9)
O1	0.0449 (11)	0.0473 (10)	0.0343 (10)	0.0153 (8)	0.0057 (8)	0.0172 (8)
O2	0.0484 (12)	0.0651 (12)	0.0400 (11)	0.0298 (10)	0.0115 (9)	0.0202 (9)
O3	0.0556 (13)	0.0830 (15)	0.0450 (12)	0.0379 (11)	0.0118 (10)	0.0319 (11)
O4	0.0734 (14)	0.0374 (10)	0.0352 (10)	0.0190 (10)	−0.0022 (9)	0.0048 (8)
O5	0.0758 (15)	0.0501 (11)	0.0541 (13)	0.0339 (11)	−0.0017 (11)	0.0066 (10)
O6	0.0979 (19)	0.0536 (13)	0.0698 (16)	0.0384 (13)	−0.0042 (14)	0.0101 (12)
O7	0.0637 (14)	0.0658 (13)	0.0455 (12)	0.0378 (11)	0.0081 (10)	0.0099 (10)
C1	0.0446 (17)	0.0557 (17)	0.0445 (16)	0.0189 (14)	0.0037 (13)	0.0240 (14)
C2	0.0445 (17)	0.073 (2)	0.0372 (16)	0.0203 (15)	0.0019 (13)	0.0206 (14)
C3	0.0428 (16)	0.0593 (17)	0.0341 (15)	0.0121 (13)	0.0033 (12)	0.0059 (13)
C4	0.0359 (14)	0.0481 (15)	0.0326 (14)	0.0140 (12)	0.0069 (11)	0.0061 (11)
C5	0.0472 (17)	0.0373 (14)	0.0472 (17)	0.0105 (12)	0.0018 (13)	0.0004 (12)
C6	0.0507 (18)	0.0360 (14)	0.0527 (18)	0.0137 (12)	0.0106 (14)	0.0120 (12)
C7	0.0374 (15)	0.0397 (13)	0.0389 (14)	0.0156 (11)	0.0095 (11)	0.0137 (11)
C8	0.0483 (17)	0.0454 (15)	0.0453 (16)	0.0220 (13)	0.0079 (13)	0.0186 (13)
C9	0.0470 (16)	0.0533 (16)	0.0353 (15)	0.0226 (13)	0.0025 (12)	0.0149 (12)
C10	0.0450 (16)	0.0419 (14)	0.0305 (14)	0.0165 (12)	0.0009 (11)	0.0060 (11)
C11	0.0328 (13)	0.0339 (12)	0.0311 (13)	0.0123 (10)	0.0077 (10)	0.0108 (10)
C12	0.0325 (14)	0.0392 (13)	0.0313 (13)	0.0140 (11)	0.0087 (10)	0.0121 (11)
C13	0.0411 (15)	0.0335 (12)	0.0311 (14)	0.0101 (11)	0.0032 (11)	0.0053 (10)
C14	0.0375 (14)	0.0314 (12)	0.0322 (13)	0.0129 (10)	0.0028 (11)	0.0076 (10)
C15	0.0393 (15)	0.0395 (13)	0.0335 (14)	0.0145 (11)	0.0019 (11)	0.0073 (11)
C16	0.0502 (17)	0.0446 (14)	0.0340 (15)	0.0134 (13)	0.0085 (12)	0.0146 (12)
C17	0.0439 (16)	0.0373 (14)	0.0450 (16)	0.0111 (12)	0.0139 (13)	0.0062 (12)
C18	0.0424 (16)	0.0465 (15)	0.0593 (19)	0.0220 (13)	0.0101 (14)	0.0178 (14)
C19	0.0480 (17)	0.0418 (14)	0.0414 (15)	0.0178 (12)	0.0073 (12)	0.0178 (12)
C20	0.0472 (16)	0.0398 (14)	0.0365 (14)	0.0175 (12)	0.0123 (12)	0.0105 (12)
C21	0.0436 (16)	0.0379 (13)	0.0368 (15)	0.0152 (12)	0.0128 (12)	0.0042 (11)
C22	0.0513 (18)	0.0398 (14)	0.0523 (18)	0.0204 (13)	0.0135 (14)	0.0085 (13)
C23	0.0539 (19)	0.0403 (15)	0.0510 (18)	0.0095 (14)	0.0103 (15)	−0.0047 (13)
C24	0.0436 (17)	0.0597 (18)	0.0361 (15)	0.0135 (14)	0.0096 (12)	0.0015 (13)
C25	0.0532 (18)	0.0582 (17)	0.0360 (16)	0.0221 (14)	0.0096 (13)	0.0108 (13)
C26	0.0548 (18)	0.0393 (14)	0.0343 (15)	0.0154 (13)	0.0107 (12)	0.0076 (11)

### Geometric parameters (Å, °)

**Table:**

Zn—O1	2.0155 (18)	C6—C7	1.432 (4)
Zn—O4	2.0325 (19)	C6—H6	0.9300
Zn—O7	2.109 (2)	C7—C11	1.405 (4)
Zn—N1	2.130 (2)	C7—C8	1.412 (4)
Zn—N2	2.126 (2)	C8—C9	1.358 (4)
Cl1—C17	1.741 (3)	C8—H8	0.9300
Cl2—C24	1.743 (3)	C9—C10	1.399 (4)
N1—C1	1.333 (3)	C9—H9	0.9300
N1—C12	1.360 (3)	C10—H10	0.9300
N2—C10	1.330 (3)	C11—C12	1.442 (3)
N2—C11	1.361 (3)	C13—C14	1.487 (4)
O1—C13	1.276 (3)	C14—C19	1.400 (4)
O2—C13	1.259 (3)	C14—C15	1.410 (4)
O3—C15	1.344 (3)	C15—C16	1.396 (4)
O3—H3A	0.9554	C16—C17	1.373 (4)
O4—C20	1.261 (3)	C16—H16	0.9300
O5—C20	1.258 (3)	C17—C18	1.387 (4)
O6—C22	1.347 (4)	C18—C19	1.376 (4)
O6—H6A	0.9509	C18—H18	0.9300
O7—H7A	0.8559	C19—H19	0.9300
O7—H7B	0.9565	C20—C21	1.496 (4)
C1—C2	1.391 (4)	C21—C26	1.401 (4)
C1—H1	0.9300	C21—C22	1.407 (4)
C2—C3	1.366 (4)	C22—C23	1.397 (4)
C2—H2	0.9300	C23—C24	1.376 (5)
C3—C4	1.410 (4)	C23—H23	0.9300
C3—H3	0.9300	C24—C25	1.378 (4)
C4—C12	1.408 (4)	C25—C26	1.377 (4)
C4—C5	1.426 (4)	C25—H25	0.9300
C5—C6	1.351 (4)	C26—H26	0.9300
C5—H5	0.9300		
			
O1—Zn—O4	105.38 (8)	N2—C10—H10	118.6
O1—Zn—O7	99.35 (8)	C9—C10—H10	118.6
O4—Zn—O7	90.95 (8)	N2—C11—C7	123.1 (2)
O1—Zn—N2	107.32 (8)	N2—C11—C12	117.3 (2)
O4—Zn—N2	87.64 (8)	C7—C11—C12	119.6 (2)
O7—Zn—N2	152.69 (9)	N1—C12—C4	123.3 (2)
O1—Zn—N1	98.75 (8)	N1—C12—C11	117.3 (2)
O4—Zn—N1	154.83 (9)	C4—C12—C11	119.4 (2)
O7—Zn—N1	92.12 (9)	O2—C13—O1	123.9 (3)
N2—Zn—N1	78.33 (8)	O2—C13—C14	118.5 (2)
C1—N1—C12	117.5 (2)	O1—C13—C14	117.6 (2)
C1—N1—Zn	128.83 (19)	C19—C14—C15	117.9 (2)
C12—N1—Zn	113.36 (16)	C19—C14—C13	121.7 (2)
C10—N2—C11	117.9 (2)	C15—C14—C13	120.3 (2)
C10—N2—Zn	128.55 (17)	O3—C15—C16	117.1 (2)
C11—N2—Zn	113.47 (16)	O3—C15—C14	122.9 (2)
C13—O1—Zn	122.31 (18)	C16—C15—C14	120.0 (3)
C15—O3—H3A	101.5	C17—C16—C15	119.7 (3)
C20—O4—Zn	130.02 (18)	C17—C16—H16	120.1
C22—O6—H6A	102.5	C15—C16—H16	120.1
Zn—O7—H7A	99.6	C16—C17—C18	121.7 (3)
Zn—O7—H7B	98.8	C16—C17—Cl1	119.1 (2)
H7A—O7—H7B	100.6	C18—C17—Cl1	119.2 (2)
N1—C1—C2	123.3 (3)	C19—C18—C17	118.5 (3)
N1—C1—H1	118.3	C19—C18—H18	120.8
C2—C1—H1	118.3	C17—C18—H18	120.8
C3—C2—C1	119.1 (3)	C18—C19—C14	122.2 (3)
C3—C2—H2	120.4	C18—C19—H19	118.9
C1—C2—H2	120.4	C14—C19—H19	118.9
C2—C3—C4	120.1 (3)	O5—C20—O4	123.9 (3)
C2—C3—H3	120.0	O5—C20—C21	118.7 (2)
C4—C3—H3	120.0	O4—C20—C21	117.4 (2)
C12—C4—C3	116.7 (3)	C26—C21—C22	117.6 (3)
C12—C4—C5	119.4 (2)	C26—C21—C20	121.1 (2)
C3—C4—C5	124.0 (3)	C22—C21—C20	121.3 (3)
C6—C5—C4	121.3 (3)	O6—C22—C23	117.3 (3)
C6—C5—H5	119.4	O6—C22—C21	122.3 (3)
C4—C5—H5	119.4	C23—C22—C21	120.4 (3)
C5—C6—C7	121.0 (3)	C24—C23—C22	119.1 (3)
C5—C6—H6	119.5	C24—C23—H23	120.4
C7—C6—H6	119.5	C22—C23—H23	120.4
C11—C7—C8	116.8 (2)	C23—C24—C25	122.3 (3)
C11—C7—C6	119.3 (2)	C23—C24—Cl2	118.2 (2)
C8—C7—C6	123.9 (2)	C25—C24—Cl2	119.5 (3)
C9—C8—C7	119.9 (3)	C26—C25—C24	118.1 (3)
C9—C8—H8	120.0	C26—C25—H25	120.9
C7—C8—H8	120.0	C24—C25—H25	120.9
C8—C9—C10	119.4 (3)	C25—C26—C21	122.4 (3)
C8—C9—H9	120.3	C25—C26—H26	118.8
C10—C9—H9	120.3	C21—C26—H26	118.8
N2—C10—C9	122.8 (2)		

### Hydrogen-bond geometry (Å, °)

**Table:**

*D*—H···*A*	*D*—H	H···*A*	*D*···*A*	*D*—H···*A*
O3—H3A···O2	0.95	1.66	2.559 (3)	155
O6—H6A···O5	0.95	1.72	2.595 (3)	151
O7—H7A···O2	0.86	1.93	2.707 (3)	150
O7—H7B···O5	0.96	1.75	2.674 (3)	163

## References

[bb1] Allen, F. H. (2002). *Acta Cryst.* B**58**, 380–388. 10.1107/s0108768102003890 12037359

[bb2] Altomare, A., Cascarano, G., Giacovazzo, C. & Guagliardi, A. (1993). *J. Appl. Cryst.* **26**, 343–350.

[bb3] Deisenhofer, J. & Michel, H. (1989). *EMBO J.* **8**, 2149–2170. 10.1002/j.1460-2075.1989.tb08338.x PMC401143 2676514

[bb4] Farrugia, L. J. (1997). *J. Appl. Cryst.* **30**, 565.

[bb5] Farrugia, L. J. (1999). *J. Appl. Cryst.* **32**, 837–838.

[bb6] Higashi, T. (1995). *ABSCOR* Rigaku Corporation, Tokyo, Japan.

[bb7] Malamatari, D. A., Hitou, P., Hatzidimitriou, A. G., Inscore, F. E., Gourdon, A., Kirk, M. L. & Kessissoglou, D. P. (1995). *Inorg. Chem.* **34**, 2493–2494.

[bb8] Maroszová, J., Martiška, L., Valigura, D., Koman, M. & Glowiak, T. (2006). *Acta Cryst.* E**62**, m1164–m1166.

[bb9] Rigaku (1998). *PROCESS-AUTO* Rigaku Corporation, Tokyo, Japan.

[bb10] Rigaku/MSC (2002). *CrystalStructure* Rigaku/MSC, The Woodlands, Texas, USA.

[bb11] Sheldrick, G. M. (2008). *Acta Cryst.* A**64**, 112–122. 10.1107/S0108767307043930 18156677

[bb12] Wen, D., Ta, H., Zhong, C., Xie, T. & Wu, L. (2007). *Acta Cryst.* E**63**, m2446–m2447.

[bb13] Wen, D. & Ying, S. (2007). *Acta Cryst.* E**63**, m2407–m2408.

[bb14] Yang, Q., Zhang, L. & Xu, D.-J. (2006). *Acta Cryst.* E**62**, m2678–m2680.

[bb15] Zhang, B.-Y., Nie, J.-J. & Xu, D.-J. (2008). *Acta Cryst.* E**64**, m937. 10.1107/S1600536808018126 PMC2961672 21202790

